# Urea assimilation and oxidation support activity of phylogenetically diverse microbial communities of the dark ocean

**DOI:** 10.1093/ismejo/wrae230

**Published:** 2024-11-12

**Authors:** Nestor Arandia-Gorostidi, Alexander L Jaffe, Alma E Parada, Bennett J Kapili, Karen L Casciotti, Rebecca S R Salcedo, Chloé M J Baumas, Anne E Dekas

**Affiliations:** Department of Earth System Science, Stanford University, 473 Via Ortega, Stanford, CA 94305, United States; Department of Marine Biology and Oceanography, Institut de Ciències del Mar, CSIC, Passeig Marítim de la Barceloneta, 37-49, 08003, Barcelona, Spain; Department of Earth System Science, Stanford University, 473 Via Ortega, Stanford, CA 94305, United States; Department of Earth System Science, Stanford University, 473 Via Ortega, Stanford, CA 94305, United States; Department of Earth System Science, Stanford University, 473 Via Ortega, Stanford, CA 94305, United States; Department of Earth System Science, Stanford University, 473 Via Ortega, Stanford, CA 94305, United States; Oceans Department, Stanford University, 473 Via Ortega, Stanford, CA 94305, United States; Department of Earth System Science, Stanford University, 473 Via Ortega, Stanford, CA 94305, United States; Department of Earth System Science, Stanford University, 473 Via Ortega, Stanford, CA 94305, United States; Department of Earth System Science, Stanford University, 473 Via Ortega, Stanford, CA 94305, United States

**Keywords:** urea, nitrogen, nitrification, chemoautotrophy, urec, metagenomics, nanosims, deep sea, mesopelagic, bathypelagic

## Abstract

Urea is hypothesized to be an important source of nitrogen and chemical energy to microorganisms in the deep sea; however, direct evidence for urea use below the epipelagic ocean is lacking. Here, we explore urea utilization from 50 to 4000 meters depth in the northeastern Pacific Ocean using metagenomics, nitrification rates, and single-cell stable-isotope-uptake measurements with nanoscale secondary ion mass spectrometry. We find that on average 25% of deep-sea cells assimilated urea-derived N (60% of detectably active cells), and that cell-specific nitrogen-incorporation rates from urea were higher than that from ammonium. Both urea concentrations and assimilation rates relative to ammonium generally increased below the euphotic zone. We detected ammonia- and urea-based nitrification at all depths at one of two sites analyzed, demonstrating their potential to support chemoautotrophy in the mesopelagic and bathypelagic regions. Using newly generated metagenomes we find that the *ureC* gene, encoding the catalytic subunit of urease, is found within 39% of deep-sea cells in this region, including the *Nitrososphaeria* (syn., *Thaumarchaeota*; likely for nitrification) as well as members of thirteen other phyla such as *Proteobacteria*, *Verrucomicrobia*, *Plantomycetota*, *Nitrospinota*, and *Chloroflexota* (likely for assimilation). Analysis of public metagenomes estimated *ureC* within 10–46% of deep-sea cells around the world, with higher prevalence below the photic zone, suggesting urea is widely available to the deep-sea microbiome globally. Our results demonstrate that urea is a nitrogen source to abundant and diverse microorganisms in the dark ocean, as well as a significant contributor to deep-sea nitrification and therefore fuel for chemoautotrophy.

## Introduction

Nitrogen (N) is an essential nutrient for all living organisms [1]; however, bioaccessible N can be a scarce and therefore limiting element in marine environments [2]. Ammonium and nitrate are among the most important forms of nitrogen in the oceans. While ammonium is assimilable by most microorganisms, nitrate must be enzymatically reduced to ammonium before assimilation, incurring an energetic cost and excluding organisms without this enzymatic machinery [3, 4]. Ammonium is therefore typically preferred, and is generally scarce below the euphotic zone (low nM range) [5] while nitrate concentrations can be orders of magnitude higher, especially at depth [6, 7]. Some microorganisms also use inorganic nitrogen as electron acceptors or donors in respiratory processes, increasing the demand for nitrogen in the environment. For example, ammonia can be oxidized to nitrite by chemoautotrophic ammonia-oxidizing archaea (AOA; i.e. *Nitrososphaeria*, syn., *Thaumarchaeota*) and ammonia-oxidizing bacteria (AOB) [8, 9]. N use in general, and ammonium use in particular, connects closely with carbon cycling, as its availability can influence rates of both heterotrophic [10] and photo/chemo-autotrophic activity [11–13].

N dynamics have been studied extensively in the euphotic zone (e.g. [5, 14]), but less is known about nitrogen cycling in the deep sea, a region increasingly recognized as hosting a diverse, active, and influential microbiome [7, 15, 16]. Urea, a form of organic nitrogen which can be cleaved enzymatically to create two molecules of ammonia, has been proposed as a key substrate for both anabolism and nitrification in the deep sea [17, 18]. As a source of energy for chemoautotrophy, urea-based nitrification could support organic matter production at depth, thereby ameliorating current discrepancies in the oceanic carbon cycle [19]. However, experimental evidence regarding the abundance [20, 21] and use of urea in the meso- and bathypelagic is still rare or lacking, respectively. *Nitrososphaeria*-affiliated *ureC* genes and transcripts (encoding the catalytic subunit of urease) have been detected in the epipelagic [17], mesopelagic [19, 22–24] and bathypelagic [15, 25], suggesting the ability of nitrifying archaea to utilize this substrate through the entire water column. Supporting this, urea-based nitrification has been measured at the ocean surface [17], at the base of the epipelagic (at 150 m [26, 27]), and within the mesopelagic (to 300 m [24] and to 1000 m [28])—at rates comparable to those for ammonia. Similarly, urea assimilation is extensive in the surface ocean [29], and has been implicated in the mesopelagic based on the observation of urea degradation at rates exceeding calculated N demand for nitrification [30]. However, direct measurements of urea assimilation have not been made in the mesopelagic or bathypelagic, measurements of urea oxidation are missing in the bathypelagic, and the prevalence and phylogenetic diversity of organisms containing *ureC* in the aphotic ocean have not been determined. Therefore, whether the ability to cleave urea is common or rare in the deep sea, taxonomically or numerically, is still unknown, and leaves the accessibility of this potentially large source of nitrogen and energy unconstrained.

In this work, we assessed the role of urea in sustaining microbial biomass production and nitrification from 50 to 4000 m water depth in the northeast Pacific Ocean. We start with an investigation of urea concentrations with depth at six sites across a 300 km transect. At two of these sites, one at the base of the continental slope (“Slope site”) and one at the far end of the transect (“Open Ocean site”), we use incubation experiments with ^13^C^15^N-urea and single-cell analysis by nanoscale secondary ion mass spectrometry (nanoSIMS) to determine the proportion of cells assimilating urea-derived nitrogen, and at what rates. We use these same incubations to determine urea- and ammonia- based nitrification rates to assess their role in microbial catabolism throughout the water column. Indeed, although genomic evidence for ammonia-based nitrification at depth is convincing [16, 31], even ammonia-based nitrification has not been experimentally confirmed below the mesopelagic. We generated thirteen deeply sequenced metagenomes throughout the Slope and Open Ocean sites, and together with public metagenomes from around the world assess the distribution of the *ureC* gene and the potential role of specific taxa in the utilization and recirculation of urea. Finally, we used the combined ammonia- and urea-based nitrification rates to estimate deep-sea carbon fixation rates, and compare these to estimated rates of sinking particulate organic carbon (POC) to estimate the significance of nitrification-based chemoautotrophy at these sites. Together, our lines of inquiry demonstrate the use of urea-derived nitrogen in both microbial anabolism and catabolism in the deep northeastern Pacific Ocean, with implications for nitrogen and carbon cycling globally.

## Material and methods

### Sample collection

**Figure 1:**
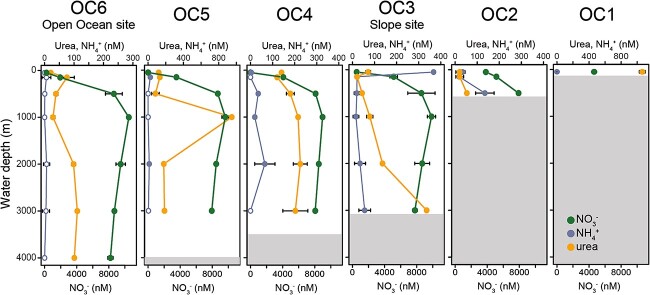
Concentrations of urea, ammonium, and nitrate for each site and depth. Note that the scale for nitrate is different from the other nutrients. Error bars indicate standard deviation of triplicate measurements. Empty dots in ammonium indicate a concentration below the detection limit. Ammonium and nitrate data are re-plotted from Arandia-Gorostidi et al., 2023. Grey area indicates sea-floor depth.

Seawater was collected in the northeast Pacific Ocean, off the coast of San Francisco north of Monterey Bay (Fig. 1, Fig. S1, Table S1), onboard the R/V *Oceanus* in March 2017. Seawater was collected using 12-liter Niskin bottles at six sites along a 300 km transect (OC1, OC2, OC3, OC4, OC5, and OC6). Samples were acquired at 50 m (all sites), 150 m (five sites), 500 m (five sites), 1000 m (four sites), 2000 m (four sites), 3000 m (four sites), and 4000 m (one site), as the water depth allowed, for a total of 29 unique site/depths. Two to four Niskin bottles were collected at each unique site/depth. Physicochemical water properties of temperature, conductivity, pressure, and fluorescence were determined with a CTD (SeaBird, USA).

### Quantification of nitrogen species

Seawater (~24 ml) from each of the 29 unique site/depths was filtered through 0.2 μm polycarbonate filters (Isopore) and stored at −80°C until analysis on shore. Urea concentration was determined following a colorimetric method [32], using 12-h incubation times at room temperature in duplicate. The detection limit was calculated to be 50 nM. Ammonium, nitrate, and nitrite concentrations were reported in a previous study [7].

### Seawater incubations with stable isotopes

Seawater from all depths sampled at site OC3 (the “Slope site”) and OC6 the “Open Ocean site”) was used for incubations with stable isotope-labelled substrates (13 unique site/depths). In one set of incubations, as previously described [7], we amended with 50 nM of ^15^N-labeled ammonium chloride (99% ^15^N, Cambridge Isotope Laboratories, USA) to investigate overall microbial activity. In another set of incubations, newly reported in this study, we amended with 50 nM of ^13^C^15^N-labeled urea (99% ^13^C and 98% ^15^N, Cambridge Isotope Laboratories, USA). Purity of the ^15^N-urea was confirmed by the manufacturer using proton nuclear magnetic resonance (^1^H-NMR) spectroscopy. For both sets, seawater was incubated in the dark in 2 or 4 L polycarbonate bottles at 10.5°C (for samples between 50 m and 150 m depth) or 4°C (samples from 500 m to 4000 m), in duplicate. Subsamples of 100–250 ml were taken from each incubation at 0 and 72 h and fixed using 3% formaldehyde overnight at 4°C. Fixed cells were filtered onto polycarbonate filters (25 mm diameter, 0.2 μm pore size; GTTP type, Millipore). Filtered, fixed cells were washed with PBS, 1:1 PBS:EtOH, and EtOH before storage at −80°C for nanoSIMS analysis. Additionally, a portion (2 × 15 ml ) of the incubated seawater was filtered through polycarbonate filters (25 mm diameter, 0.2 μm pore size; GTTP type, Millipore) into 50 ml Falcon tubes at 0, 24, and 72 h. The filtrate was stored at −80°C until nitrification analysis. Further information about the experimental setup is described in our previous publication [7].

### Single-cell isotope uptake by nanoSIMS

Single-cell uptake rates for ^13^C and ^15^N were analyzed by nanoscale secondary ion mass spectrometry (nanoSIMS) using a NanoSIMS 50 L (CAMECA, Gennevilliers, France) housed in the Stanford Nano Facility. Analysis conditions are described in the Supplemental Material. Between 61 and 128 cells were analyzed from a single incubation for each of the 13 unique site/depths from the Slope and Open Ocean sites. The isotope images were analyzed using LANS software [33], resulting in the quantitative analysis of isotopic ratios of ^13^C^−12^C^−^/^12^C_2_^−^ and ^12^C^15^N^−^/^12^C^14^N^−^. To determine cell-specific isotope ratios, the ^12^C^14^N^−^ channel was used to manually draw regions of interest with outlines just inside the cells. Cells were considered isotopically enriched and therefore consumers of a particular substrate if their isotope ratio was greater than 2 standard deviations above the mean isotope ratio of the 0 h cells from each site [7, 34]. The isotope-based growth (Ka), relative to the initial N and C content, and the single-cell assimilation rates in fg cell^−1^ h^−1^ were calculated following equations published previously [35]. The integrated rates for the epi-, meso-, and bathypelagic regions were calculated as previously described [7]; multiplying cell density at each depth with the cell-specific assimilation rates (in fg cell^−1^ h^−1^) and the total volume of each region. Statistical differences between the assimilation rates of each substrate were calculated using the Wilcoxon test in R (R version 4.1.3).

### Nitrification rates

Nitrification rates were determined from the rate of production of ^15^N-labeled NOx (NO_3_^−^ + NO_2_^−^) in incubations with ^15^N-ammonium and ^15^N-urea at both Slope and Open Ocean sites between 150 m and 4000 m depths. 50 m samples could not be analyzed due to the low concentration of NOx at this depth. Incubation subsamples were collected as described above, and the 72-h time points were measured in duplicate. The ^15^N/^14^N ratio of NOx was determined by isotope ratio mass spectrometry using the denitrifier method [36, 37] in the Stanford Stable Isotope Biogeochemistry Laboratory and calibrated using parallel analyses of nitrate isotope reference materials USGS32, USGS34, and USGS35 [38]. The nitrification rates were determined using a linear fit of ^15^N-NOx over time in each incubation [39].

### Estimation of gravitational POC flux

The gravitational Particulate Organic Carbon flux (POC_flux_) at 100 m depth was estimated by multiplying the net primary production by the carbon export efficiency (*e-eff*) calculated according to the following Equation 1 [40]:


(1) \begin{equation*} e\!-\!eff=0.23\ \times\ {\mathrm{e}}^{\left(-0.08\ \times \ \mathrm{SST}\right)} \end{equation*}

where SST is the Sea Surface Temperature. Satellite-derived net primary production was downloaded from Ocean productivity site (http://sites.science.oregonstate.edu/ocean.productivity/index.php) as 8 days file format treated from the VGPM algorithm [41].

### DNA extraction and metagenomic sequencing

Samples for metagenomic analysis were collected at all depths at the Slope site and the Open Ocean site. Seawater (5–25 L, depending on depth and, therefore, cell density) was filtered through 0.2 μm Sterivex filter units (Millipore, Germany) and flash-frozen in liquid N_2_ immediately after collection. DNA was extracted using the AllPrep DNA/RNA kit (Qiagen, Valencia, CA, USA), following the manufacturer’s protocol. DNA concentration was measured using the Quant-iT PicoGreen dsDNA Reagent (Invitrogen, Carlsbad, CA, USA). To account for low yield from some deep-sea samples, extractions were normalized to 8 ng and processed using the Takara ThruPLEX kit (Takara Biosciences USA, Mountain View, CA) followed by 10 PCR cycles. Size selection was performed using the PippinHT (Sage Science, Beverly, MA). Finally, prepared metagenomic libraries were sequenced using the NovaSeq S4 PE150 platform (Illumina) at UC Davis sequencing facility (California, USA) with a target sequencing depth of ~45 gigabase pairs per sample.

### Metagenomic analysis and binning

Paired-end reads were pre-processed with bbduk to remove adapters and to trim low-quality sequences. Trimmed reads were assembled individually using MEGAHIT (v1.2.9) [42, 43]. Metagenomic assemblies accounted for an average of ~60% of the trimmed metagenomic reads. To analyze the distribution and diversity of *ureC* genes within the OC1703 assemblies, as well as those from the public GEOTRACES, TARA Oceans, and Malaspina datasets, we first created a gold standard list of functional *ureC* proteins in cultured marine microorganisms capable of urea degradation (see Table S7). Next, we used this list to identify *ureC*-encoding contigs using a modification of the PPIT [44] R package (see Supplementary Information). The abundance of *ureC* in each sample was calculated by mapping trimmed metagenomic reads against identified *ureC*-containing contigs with bbmap using default parameters (V39.01) [45] and quantifying these reads (in terms of reads per kilobase million mapped, or RPKM) with the samtools package using the flagstat output (V1.17) [46]. The abundance of *recA*, *amoA*, and nitrate-related genes were calculated using the same approach, though with a modified initial annotation step (Supp. Info). To estimate the proportion of cells containing *ureC*, we normalized its relative abundance to that of *recA*, taking into account the average *ureC* gene copy number per genome observed in the set of metagenome-assembled genomes (MAGs) resolved using the methodology described below (1.1 *ureC* genes/MAG). Additionally, we analyzed *ureC* genetic diversity using a BLAST-based approach that assigns a putative taxonomy to contigs based on the consensus of all genes it encodes (Supp. Info.). The relative abundance of each *ureC*-encoding contig for which taxonomy could be assigned was computed by dividing its sequencing coverage by the total coverage of all *ureC*-encoding contigs in that sample (≥50% breadth). We also subjected the newly generated OC1703 assemblies to metagenomic binning, forming a set of medium to high quality MAGs (≥50% completeness and ≤ 5% redundancy as determined by CheckM (v1.2.2) [47] for downstream analysis (Supp. Info.). These bins accounted for an average of ~28% of the trimmed metagenomic reads.

### Statistical analyses

**Figure 2:**
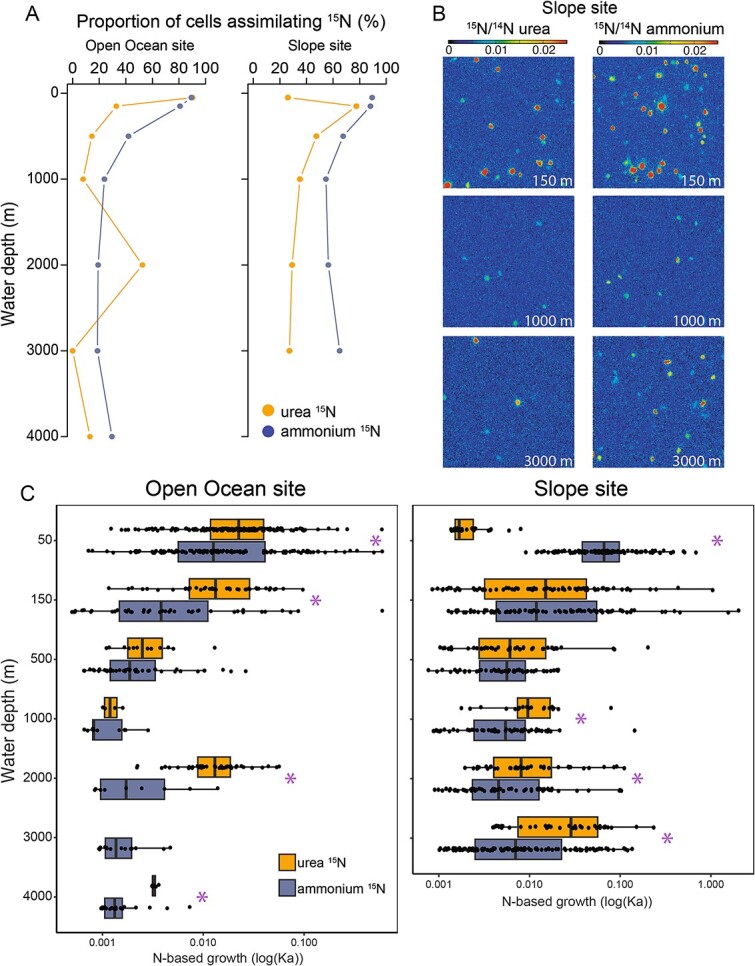
Single-cell assimilation of urea-derived N, compared to ammonium. (**A**) the depth profile plots show the proportion (%) of cells that assimilated nitrogen from urea or ammonium at each depth of the Open Ocean and slope sites. (**B**) NanoSIMS images of cells showing assimilation of ^15^N from urea and ammonium in incubations of seawater from the slope site. The color scale indicates the ^12^C^15^N^−^/^12^C^14^N^−^ ratio for the analyzed areas. (**C**) Boxplots represent the N-based growth (Ka) in logarithmic scales for individual cells from both urea- and ammonium-amended incubations at each depth of the Open Ocean site (left) and slope site (right). Purple asterisks indicate that the difference between ammonium and urea rates are significant (ANOVA, *P* value <0.05).

To test the differences in isotopic assimilation (Fig. 2C), the Wilcoxon signed-rank test was used to compare the median assimilation rates between the ^15^N-urea and ^15^N-ammonium incubations at each depth, due to the data distribution not following a normal distribution. Differences were considered significant if *P* value<0.05). To test the differences in nitrification rates between the urea and ammonium incubations, we used a *t*-test to compare the mean rates of each incubation at a given depth. A comparison of the change of *ureC* relative abundance with depth was performed by ANOVA analysis with the Tukey’s honest significant difference test correction. All the statistical tests were performed in R (R version 4.3.1).

## Results

### Quantification of nitrogen species

We detected urea in all samples, ranging from below the detection limit (50 nM) at 50 m water depth at the Open Ocean site (OC6, 281 km from shore) to 1.1 μM at 50 m water depth at OC1, the most coastal site (14 km from shore, Fig. 1). Urea concentrations were highest below the euphotic zone, and generally higher than those of ammonium. The physicochemical analysis of the sampling sites and the concentrations of ammonium and nitrate were described in a previous study [7].

### Single-cell urea uptake with depth

We detected microbial assimilation of urea-derived N in all depths at both the Slope and Open Ocean sites except one (Open Ocean site, 3000 m water depth, Fig. 2). We determined the proportion of cells assimilating urea-derived N as well as the magnitude of incorporation (per cell and the average for all cells assimilating it) and compared to the values we previously determined for ammonium assimilation in the same samples [7]. In the Open Ocean site, the highest proportion of cells incorporating urea-derived ^15^N occurred at 50 m (90%; Fig. 2A)—almost exactly the same as for ammonium (89%). The proportion of cells incorporating urea-derived N generally decreased with depth, similar to the trend for ammonium, with an exception at 2000 m where the proportion spiked to 53%. The trend in the Slope site was different from the Open Ocean site, with the lowest proportion of cells incorporating urea-derived N at 50 m (26%) and the highest at 150 m (78%). The proportions in the meso- and the bathypelagic region remained relatively high (37% on average) at the Slope site, higher than at the same depths at the Open Ocean site (18% on average). The trends in the average N-assimilation rates from urea followed the same pattern as the trends in the portion of cells assimilating urea (Fig. 2C). Overall, the Slope site had a higher proportion of cells assimilating urea-N and at higher rates than at the Open Ocean site.

On average, urea-N was assimilated at higher single-cell rates than ammonium. For the cells assimilating these substrates, the mean single-cell assimilation rates in the Slope site were statistically significantly higher for urea than ammonium at 1000 m, 2000 m, and 3000 m depth (Wilcoxon test, *P* value <0.05), with an average of 83% higher assimilation rates for urea. The difference between urea and ammonium uptake was even more pronounced at the Open Ocean site, with statistically significantly higher mean incorporation rates of urea at 50 m, 150 m, 2000 m, and 4000 m depth (Wilcoxon test, *P* value <0.05), and an average assimilation rate an order of magnitude higher for urea than ammonium.

We detected assimilation of urea-derived ^13^C (Fig. S2). In the Slope site, cells assimilating urea-derived ^13^C were only detected in the epipelagic region and at 3000 m depth (up to ~20% of cells), while in the Open Ocean site, cells assimilating urea-derived ^13^C were detected throughout the water column (~25% at 500 m depth and ~20% at 2000 m and 4000 m depth). At the Open Ocean site, we found that cell-specific ^13^C-urea assimilation was lower than that for ^15^N in the epipelagic region, but that ^13^C-urea assimilation surpassed that for ^15^N in the meso- and bathypelagic regions. In general, while single-cell rates of urea-derived ^15^N assimilation showed variable trends with depth, those for urea-derived ^13^C increased with depth.

### Ammonia- and urea-based nitrification

**Figure 3:**
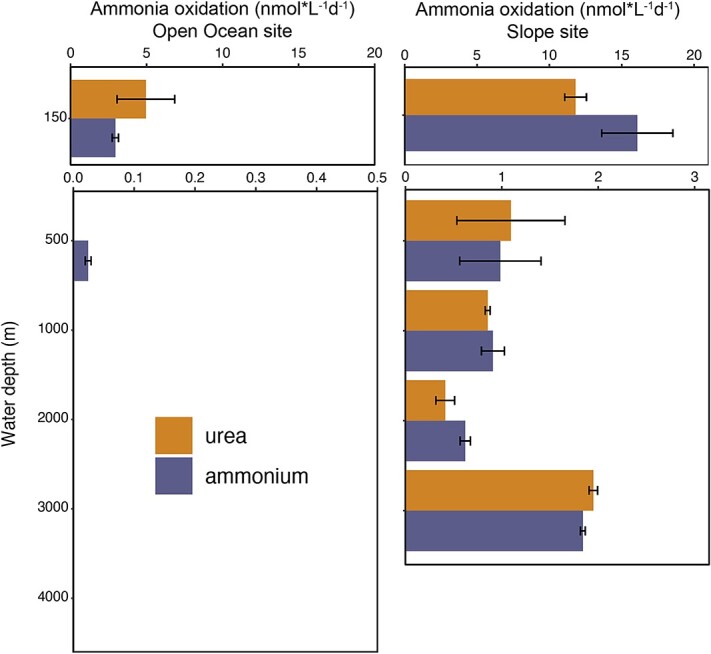
Urea- and ammonium-based ammonia oxidation rates at each depth of the slope site and Open Ocean site. While at the slope site ammonia oxidation was detected at all depths, for the Open Ocean site no significant ammonia oxidation was detected below 150 m depth (except for ammonia incubations at 500 m depth). Error bars represent standard deviation of duplicate measurements.

We determined rates of ammonia- and urea-based nitrification for all depths in the Slope and Open Ocean sites below 50 m (Fig. 3). We detected the production of ^15^N-NOx (i.e. active nitrification) in incubations with ^15^N-urea and ^15^N-ammonium at all depths in the Slope site. Rates of nitrification were highest at 150 m (16.1 and 11.8 nmol N l^−1^ for ammonia and urea, respectively), dropped by an order of magnitude by 500 m, and then were relatively consistent with depth with a slight uptick at 3000 m. At the Open Ocean site, we detected ammonia-based nitrification at 150 m and 500 m, and urea-based nitrification at 150 m, but not below. Rates of urea-based nitrification were similar to that for ammonia, and statistically indistinguishable at all depths (*t*-test >0.1 for all depths).

### Comparison of organic carbon sources to the deep sea

In order to assess the significance of deep-sea nitrification to the marine carbon cycle we estimate and compare two sources of organic carbon to the deep sea: the POC consisting of photosynthetic detritus sinking from the euphotic zone (“gravitational POC”) and the inorganic carbon fixed via nitrification-based chemoautotrophy in the deep sea. We estimated POC export fluxes to be 26 and 128 mg C m^−2^ d^−1^ at the base of the euphotic zone (100 m) at the Open Ocean and Slope sites, respectively. In parallel, assuming a DIC fixation yield of 0.09 mol C fixed mol-N^−1^ oxidized [48], we converted the rates of urea- and ammonium-based nitrification measured here into carbon fixation rates and integrated them over the dark water column (100–3000 m or 100–4000 m, depending on the site). We found rates of 1.4 and 8.8 mg C m^−2^ day^−1^ of autotrophic carbon fixation for the Open Ocean and Slope sites respectively, corresponding to 5 and 7% of the gravitational POC fluxes entering the dark ocean.

### Relative abundance and taxonomic affiliation of ureC genes

**Figure 4:**
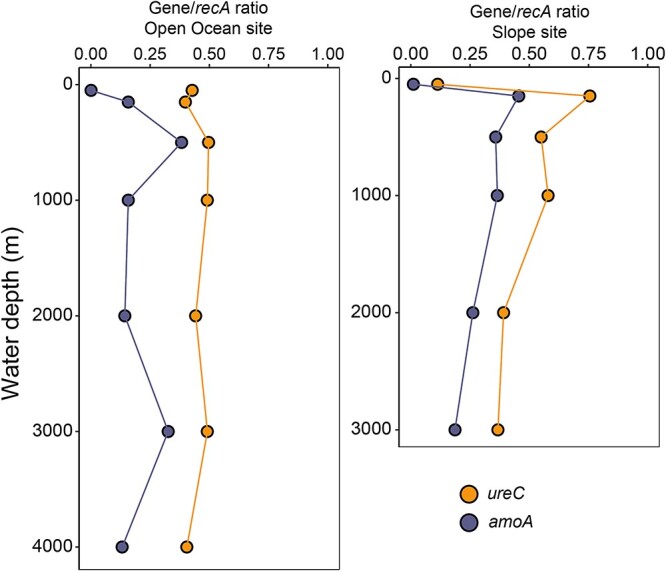
Vertical profiles showing gene abundance of *ureC* and *amoA* genes relative to a housekeeping gene (*recA*) in the metagenomes at the Open Ocean site (left) and slope site (right).

We sequenced and assembled metagenomes (average 57.8 Gb/metagenome) from all thirteen depths of the two sites where isotope incubations were conducted (Slope and Open Ocean sites; Table S2). We detected *ureC* at all depths investigated (Fig. 4). The relative abundance of *ureC* genes was lowest at 50 m (0.11 *ureC*/*recA* ratio) and highest at 150 m (0.76 *ureC*/*recA* ratio) at the Slope site, generally mirroring the trend in proportion of cells assimilating urea at this site. The prevalence of *ureC* was more consistent with depth at the Open Ocean site, with the maximum ratio (0.5 *ureC*/*recA*) found at 500 m, 1000 m, and 3000 m depths (Fig. 4). We compared the relative abundance of *ureC* genes within the entire microbial population to that of *amoA*—the gene encoding subunit A of ammonia monooxygenase, essential for archaeal nitrification—and found that *ureC* was consistently twice as abundant as *amoA* (0.45 *ureC*/*recA* and 0.23 *amoA*/*recA* on average; Fig. 4). This indicates a great potential to cleave urea outside of the AOA, i.e. in organisms with different catabolisms.

**Figure 5:**
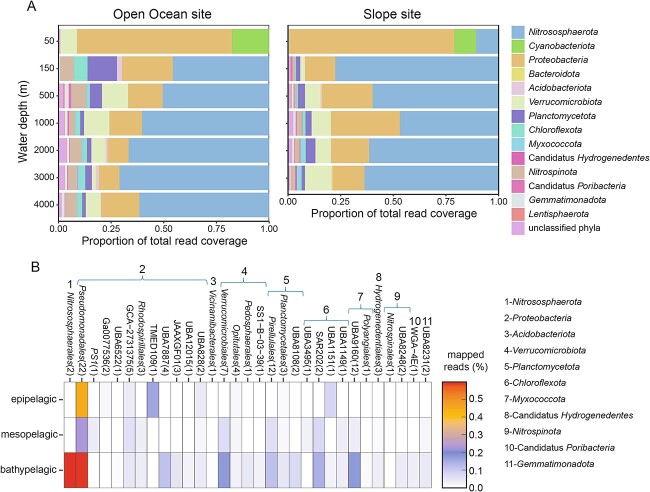
Diversity, distribution, and relative abundance of taxa containing *ureC*. (**A**) Taxonomic analysis of the *ureC*-containing contigs classified at the phylum level for each water depth, each site separately. (**B**) Relative abundance of *ureC*-containing MAGs within each order (expressed as percentage of total mapped reads) with water depth, slope and Open Ocean sites combined. Phylum affiliation is indicated. The number of MAGs within each order is indicated in parentheses.

A subset of *ureC*-encoding contigs (≥3000 bp, non-eukaryotic, Table S4) were then analyzed to determine the taxonomic composition of organisms with the genetic potential to cleave urea. These *ureC*-containing contigs were associated with diverse phylogenetic groups (fourteen distinct phyla) and showed a consistent shift with depth between sites (Fig. 5A, Table S3). In the 50 m sample, *ureC*-containing contigs were associated primarily with *Proteobacteria* at both the Open Ocean and Slope sites (73.5% and 78.4% of the *ureC*-containing community, respectively), but were also associated with *Cyanobacteria* (17.7% and 10.4%), *Nitrosophaerota* (10.7%, only in the Slope site), and *Verrucomicrobia* (7% and 0.5%). While *Cyanobacteria* were only detected at 50 m, members of the *Nitrosophaerota* comprised an increasingly large fraction of the *ureC*-containing community with depth (>50% in most samples), together with increases in members of the *Planctomycetota* (up to 14.0%), *Verrucomicrobiota (*up to 12.5%), *Nitrospinota* (up to 7.2%), and *Myxococcota* (up to 1.6%).

### Taxonomic identification and investigation of MAGs containing ureC

To complement our contig-based analyses, we generated 109 unique *ureC*-containing MAGs with ≥50% completeness and ≤ 5% redundancy (Table S5). Taxonomic identification of these *ureC*-containing MAGs indicated they were from 11 distinct phyla (Fig. 5B), capturing most groups identified in the contig-based approach. Similarly, MAG-based abundance analyses roughly recapitulated the distribution pattern of taxonomic groups with depth, with proteobacterial *ureC*-containing MAGs more relatively abundant at the surface and those of the *Nitrosophaerota* and others becoming more relatively abundant at depth. Only MAGs belonging to the alphaproteobacterial TMED109 clade and the *Chloroflexota* UBA1151 were more relatively abundant in the epipelagic region than in the deepest regions. Overall, a higher relative abundance of *ureC*-containing MAG groups was found in the bathypelagic (2.56% of all mapped reads in the bathypelagic versus 0.76% in the epipelagic). MAGs within the *Nitrososphaerales* order (phylum *Nitrososphaerota*) and the *Pseudomonadales* order (phylum *Proteobacteria*) were the most relatively abundant *ureC*-containing groups in the bathypelagic region (recruiting 0.60% and 0.59% of total mapped reads respectively). Other groups, including the *Verrucomicrobiales* and the *Myxococcota* UBA9160 orders, were exclusively found below the photic zone.

### UreC prevalence in global datasets and comparison to other genes

**Figure 6:**
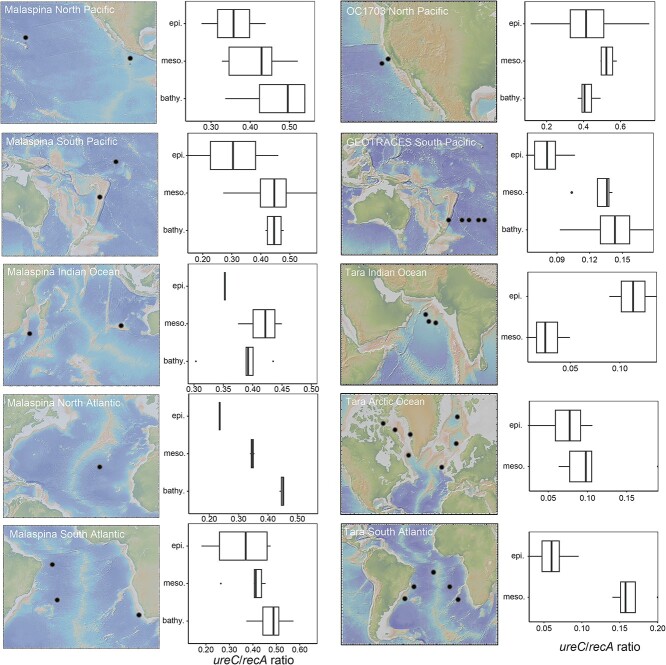
Box plots showing abundance of *ureC* genes relative to total mapped reads in the epipelagic (0–200 mbsl), mesopelagic (200–1000 mbsl), and bathypelagic (1000–4500 mbsl) regions of different ocean regions. The North Pacific Ocean is shown in the OC1703 (this study) and Malaspina datasets, Southwest Pacific Ocean in the GEOTRACES dataset, North Atlantic in the Malaspina dataset, South Atlantic, and Indian oceans in the Malaspina and Tara oceans datasets and the Arctic Ocean in the Tara oceans datasets. Gene abundances are displayed as the ratio between *ureC* and *recA* (both gene coverages calculated as RPKM). For the Tara oceans, only samples for the epipelagic and mesopelagic regions were available.

To assess the generality of our findings across the global ocean, we determined the relative abundance of *ureC* genes in epipelagic, mesopelagic, and bathypelagic depths from different ocean basins using the publicly available Tara Ocean, GEOTRACES, and Malaspina databases (Fig. 6, Table S6). Based on comparison with *recA*, we estimate that 10–46% of cells in the global deep sea contain *ureC* (median 36%), consistent with our findings in the North Pacific Ocean. Relative abundances of *ureC* increased with depth in the GEOTRACES data from the South Pacific [49], Tara Oceans data in the Arctic and South Atlantic Ocean [50, 51], and Malaspina data in the North Atlantic, South Atlantic, North Pacific, South Pacific, and Indian Oceans [16]. The only exception was found in sites from the northern Indian Ocean where a higher relative abundance of *ureC* was observed at epipelagic depths than mesopelagic depths (no bathypelagic metagenomes are available).

We also compared the distribution of *ureC* with genes involved in the metabolism of nitrate, the most abundant source of nitrogen in the deep sea, in order to compare the potential importance of both substrates. Similar to *ureC*, the prevalence of genes (measured as gene/*recA* ratio) such as *nirA* or *nasA* (key genes in assimilatory nitrate reductase), *nirB* and *nirD* (dissimilatory nitrate reduction), *nirK* and *nirS* (denitrification), as well as *nxrA* and *nxrB* (nitrite oxidoreductase) increased with depth in all analyzed datasets (OC1703, GEOTRACES and Malaspina, Fig. S3). However, the relative abundance of *ureC* was consistently higher than these other genes. This difference is particularly notable in the Malaspina dataset, which is the dataset that best reflects the gene distribution in the world oceans; there, the *ureC*/*recA* ratio exceeds the *nasA*/*recA* ratio by an order of magnitude.

## Discussion

Urea is increasingly recognized as a source of nitrogen for cell growth [29, 52, 53] and nitrification [17, 18] in the sunlit ocean. In the euphotic zone, nitrogen from urea is assimilated by phylogenetically diverse taxa, including *Cyanobacteria*, *Proteobacteria*, and *Nitrososphaerota* (e.g. *Nitrososphaeria*) [14, 18, 19, 26, 53, 54], at rates exceeding those for nitrate, leucine, glutamate [14], and even ammonium [55]; it is also oxidized by nitrifying *Nitrososphaeria* to support chemoautotrophy [56, 57]. Our observations in the northwest Pacific Ocean indicate that the significance of this molecule extends to the aphotic zone—where an equally broad yet predominantly different set of organisms cleave it—and indicate that its role may be even more central to ecosystem functioning there than in surface waters. Our data show that both the concentration of urea and the relative abundance of *ureC* genes increase with depth, as does the microbial preference for it as a nitrogen source over ammonia. Additionally, while rates of urea-based nitrification are lower at depth than at the surface, they are comparable to that of ammonia in both realms, and likely play an outsized role in microbial community dynamics at depth by supporting the production of organic matter in a more energy- and carbon-limited system than at the surface.

We found that urea-derived nitrogen is widely and extensively assimilated by microorganisms in the aphotic zone. We detect assimilation of urea-derived nitrogen in 25% of cells on average in the meso- and bathypelagic regions. However, since many cells in these regions are below our detection limit of anabolic activity [7], it is possible that we are underestimating the proportion of cells assimilating urea. Using ammonium uptake as a proxy for detectable anabolic activity [7, 58] we estimate that 60% of detectably active cells in the meso- and bathypelagic assimilate urea-derived nitrogen. Cross-feeding of ^15^N-labelled substrates can cause these proportions to be greater than the number of cells directly consuming urea, and for this reason, we refer to the assimilation of ^15^N in the ^15^N-urea incubations as assimilation of “urea-derived” nitrogen. However, the distinct trends between urea and ammonium (Fig. 2), suggest that cross-feeding was minimal and direct use of urea is the dominant process underlying our observations. Additionally, the paired metagenomic data is roughly consistent with the uptake data; we estimate that an average of 39% of cells in the meso- and bathypelagic at these sites contain a *ureC* gene (as described in more detail below). Regardless of what proportion of the assimilation was directly from urea versus recycled substrates, the widespread and high rates of consumption of urea-derived N indicates that the large reservoir of urea-nitrogen in the deep sea—on average an order of magnitude more abundant than ammonium—is available to most cells.

Nitrate remains the largest pool of fixed nitrogen in the deep sea, averaging over two orders of magnitude more abundant than urea at our study sites. Our observations of urea assimilation occurred in the presence of these high concentrations of nitrate, suggesting a preference for urea over nitrate. Indeed, we found that *ureC* genes were more abundant than those related to assimilatory nitrate reduction (such as *nasA*) within our study sites, as well as a broad distribution of publicly available deep sea datasets (Fig. S3). Previous work has also reported relatively low detection of *nasA* in the Malaspina global deep-sea metagenomic dataset [15]. Preference for urea is likely related to the higher energy requirements of the assimilatory reduction of nitrate [59, 60], a difference that might be particularly relevant in the energy-poor aphotic zone. While gene abundances are useful indicators of potential activity, and how well a given ability is distributed across a community, direct comparisons of the uptake of nitrate and urea in the deepest regions of the oceans would be beneficial to directly compare the proportions of cells capable of assimilating each, and with what preference. The meso- and bathypelagic regions accounted for nearly half of the total pelagic urea assimilation in the Slope site—more than it contributed to either ammonium or amino acid assimilation [7]—indicating that urea is a more important nitrogen source in the deep sea relative to the surface than for either ammonium or amino acids.

Urea also represents a major potential substrate for nitrification by members of the *Nitrososphaeria* [17–19, 24]. Remarkably, urea-based nitrification can also happen in the presence of substantial ammonia [17], suggesting that urea is not only an alternative when ammonia is scarce. Furthermore, a recent study shows that some AOB repress the use of extracellular ammonia in the presence of ammonia derived from urea hydrolysis in the cytoplasm [55]. While previous studies have highlighted the significance of urea-driven nitrification in the epipelagic [17, 18, 61] and mesopelagic regions [24, 28], urea-driven nitrification has not been directly measured in the bathypelagic region. The detection of urea-based nitrification at all depths of our Slope site suggests that bathypelagic nitrifiers can indeed use urea as a substrate (Fig. 3). Oxidation of ammonia after urea hydrolysis by other community members is also possible, but even in this case, this confirms that urea-derived nitrogen is readily available to microbes for nitrification. Direct uptake and hydrolysis of urea by deep-sea nitrifiers is supported by the metagenomic analysis, which showed extensive genetic potential for urea use by nitrifiers: *ureC* genes were found within *Nitrososphaeria* MAGs and over half of sequencing coverage of *ureC*-containing contigs in the meso- and bathypelagic was affiliated with *Nitrososphaeria* (Fig. 5A). The rates of urea-based nitrification were statistically indistinguishable from those for ammonium at all depths, consistent with the previous work in the mesopelagic [24, 28], indicating a potentially significant role for urea in deep-sea nitrification. Rates of both ammonia- and urea-based nitrification decreased with depth and distance from shore (Fig. 3), consistent with the trends we observed in overall anabolic activity previously at this site [7].

Using metagenomics, we determined both the distribution of *ureC* in microbial communities at our study site and in globally sourced datasets, and also classified the taxa containing *ureC*. We detected *ureC* genes throughout the water column and found that their prevalence within the community—the proportion of microbial cells possessing it in a given sample—reached a maximum in the aphotic zone in both our study site and the other global datasets we analyzed. This is consistent with a previous analysis that found that the prevalence of *ureC* within Thaumarchaeota increased with depth in both Artic and Antarctic regions [19], as well as a recent proteomics study which observed peak relative abundance of urease in the bathypelagic region of the global ocean [62]. Overall, we see that about a third of the cells in the dark ocean (average 39% in our dataset, and average of 30% in the global datasets) contain *ureC*.

Both the contig- and MAG-based analyses identified diverse taxa containing *ureC* genes at our site, with fourteen distinct phyla identified by the former and 11 by the latter. The MAG-based taxonomic identification of *ureC*-containing genomes is more robust than the contig-based identifications due to the greater sequence length available for consideration and less vulnerability to misclassifications due to horizontal gene transfer. However, the contig-based approach provides a more comprehensive overview of the community (accounting for, on average, 32% more of the total metagenomic reads than the MAG set; Fig. S4), and includes taxa that systematically evade genomic binning. Notably, 11 of the fourteen phyla identified in the contig-based analysis were also identified with the MAG-based analysis. The groups identified as containing *ureC* in the 50 m samples are generally consistent with previous work in the euphotic zone, especially in the identification of *Gammaproteobacteria* [22] and *Prochlorococcus* [14]. The deep-sea analysis revealed that some taxonomic groups with members known to use urea at the surface also have members with the genetic capacity to do so at depth, including *Nitrososphaeria* (*Nitrososphaerales*), *Verrucomicrobiota*, and *Myxococcota*, as well as members of several groups not before reported to utilize urea, including SAR202 and alphaproteobacterial TMED109. We interpret the presence of *ureC* genes in taxa not known to oxidize ammonia, and in MAGs without an *amoA* gene, as evidence of potential urea use for nitrogen acquisition. Conversely, when found together with *amoA* (i.e. within the *Nitrososphaeria*), it may be used for both nitrogen acquisition for biomass and for nitrification. While our metagenomic analysis is consistent with a large role for urea in nitrifying organisms in the deep sea—evidenced by the large fraction of *ureC* genes within the *Nitrososphaeria*—our work also highlights the wide diversity of organisms capable of cleaving it. As not all nitrifiers contain urease (e.g. *Nitrosopelagicus brevis* CN25 [63], *Nitrosopumilus maritimus* [23]), there may be an important relationship between heterotrophic urea degraders and chemoautotrophic nitrifiers, with ammonia shared in one direction and organic carbon (and/or other metabolites [64]) in the other. This is similar to the exchange of ammonia for nitrite previously suggested between *ureC*-containing nitrite-oxidizing bacteria and archaeal nitrifiers [54].

The implications of deep-sea ammonia- and urea-based nitrification on the marine carbon cycle are considerable. It is often assumed that the main—and essentially only—source of organic carbon to the dark ocean is gravitational POC [65]. However, the persistent imbalance between known supply and demand of organic matter in the deep sea highlights the inadequacies of this perspective [66]. The potential for endogenous production of organic carbon (e.g. chemoautotrophy) to contribute significantly to the deep-sea carbon budget is increasingly recognized [67, 68], with regional measurements of dark DIC fixation equaling an estimated 15–53% of gravitational POC, and 12–72% of organic carbon demand (North Atlantic and Arctic Oceans [69–71]). Nitrite-oxidizing bacteria have been reported to contribute 15–45% of total inorganic carbon fixation in the mesopelagic North Atlantic based on microaudioradiography [54], but how specific organisms/metabolisms contribute to total chemoautotrophy is generally not well constrained. Rates of maximum potential DIC fixation by ammonia-oxidizing archaea have been previously approximated to be over an order of magnitude lower than total dark DIC fixation in the North Atlantic, but were calculated based on availability of ammonia, not direct measurements [69].

Our observations in the Northwest Pacific Ocean indicate that urea- and ammonia-based nitrification could support DIC fixation of 5 and 7% of the estimated gravitational POC entering the top of the mesopelagic at the Open Ocean and Slope sites, respectively. As total DIC fixation was not measured here, we cannot determine the contribution of these processes to total chemoautotrophy at these sites. However, these values are significant in comparison to the POC flux; gravitational POC fluxes and quality (bioaccessibility) decrease significantly with depth in the water column [72, 73]. In contrast, organic carbon generated at depth is generally labile, highlighting the potential importance of even small amounts of endogenously produced organic carbon [74]. To fully assess the role of nitrification and chemoautotrophy more generally in helping balancing the carbon budget, more studies are required, including direct measurements of carbon fixation ideally at *in situ* pressures and concurrent measurement of sinking POC flux. However, our estimates provide evidence that the combination of urea- and ammonia-based nitrification can serve as a substantial source of endogenous organic carbon in the aphotic northeastern Pacific Ocean, and supports the notion that deep-sea chemoautotrophy should not be overlooked in models of the biological carbon pump.

In summary, our study reveals a large reservoir of urea-N in the deep sea (Fig. 1), widespread genetic potential for urea utilization in the meso- and bathypelagic (Figs. 4 and 6), and direct evidence for both extensive assimilation of urea-derived nitrogen (Fig. 2) and the persistence of both urea- and ammonia-based nitrification throughout the epi-, meso-, and bathypelagic (Fig. 3). While additional direct measurements are necessary to confirm our results globally, we contend that urea use is likely widespread throughout the global deep sea on the basis of the generally physicochemically representative nature of our study site and the high proportions of *ureC*-encoding microorganisms throughout the global metagenomic datasets analyzed here. These results address long-standing hypotheses about the potential for urea to fuel nitrification in deep waters, and indicate the potential for chemoauototrophy at depth to significantly impact the marine carbon budget.

## Supplementary Material

Arandia-Gorostidi_etal_ISME_SuppInfo_Urea2_wrae230

Arandia-Gorostidi_etal_ISME_SuppTables_Urea_wrae230

## Data Availability

Read data and MAGs analyzed in this study are available through NCBI at PRJNA1054206.
